# Adolescents’ Perspectives on Personalized E-Feedback in the Context of Health Risk Behavior Screening for Primary Care: Qualitative Study

**DOI:** 10.2196/jmir.7474

**Published:** 2017-07-20

**Authors:** Garret G Zieve, Laura P Richardson, Katherine Katzman, Heather Spielvogle, Sandy Whitehouse, Carolyn A McCarty

**Affiliations:** ^1^ Center for Child Health, Behavior and Development Seattle Children’s Research Institute Seattle, WA United States; ^2^ Department of Psychology University of California, Berkeley Berkeley, CA United States; ^3^ Department of Pediatrics University of Washington Seattle, WA United States; ^4^ Division of Adolescent Medicine BC Children's Hospital University of British Columbia Vancouver, BC Canada

**Keywords:** adolescent, screening, health behavior, motivation, primary health care, software, technology, qualitative research

## Abstract

**Background:**

Electronic health screening tools for primary care present an opportunity to go beyond data collection to provide education and feedback to adolescents in order to motivate behavior change. However, there is limited research to guide feedback message development.

**Objective:**

The aim of this study was to explore youth perceptions of and preferences for receiving personalized feedback for multiple health risk behaviors and reinforcement for health promoting behaviors from an electronic health screening tool for primary care settings, using qualitative methodology.

**Methods:**

In total, 31 adolescents aged 13-18 years completed the screening tool, received the electronic feedback, and subsequently participated in individual, semistructured, qualitative interviews lasting approximately 60 min. Participants were queried about their overall impressions of the tool, perceptions regarding various types of feedback messages, and additional features that would help motivate health behavior change. Using thematic analysis, interview transcripts were coded to identify common themes expressed across participants.

**Results:**

Overall, the tool was well-received by participants who perceived it as a way to enhance—but not replace—their interactions with providers. They appreciated receiving nonjudgmental feedback from the tool and responded positively to information regarding the consequences of behaviors, comparisons with peer norms and health guidelines, tips for behavior change, and reinforcement of healthy choices. A small but noteworthy minority of participants dismissed the peer norms as not real or relevant and national guidelines as not valid or reasonable. When prompted for possible adaptations to the tool, adolescents expressed interest in receiving follow-up information, setting health-related goals, tracking their behaviors over time, and communicating with providers electronically between appointments.

**Conclusions:**

Adolescents in this qualitative study desired feedback that validates their healthy behavior choices and supports them as independent decision makers by neutrally presenting health information, facilitating goal setting, and offering ongoing technological supports.

## Introduction

Most of the leading contributors to adolescent morbidity and mortality are preventable health risk behaviors such as substance use, unprotected sexual activity, and unsafe driving practices [1]. Primary care visits present a promising opportunity to recognize and intervene with adolescents who engage in these behaviors. For example, in the United States, many adolescents aged 12-17 years attend a preventive medical visit annually, with estimates ranging from 37-82% [2-4]. When annual attendance is considered over the course of the adolescent years, primary care providers may have multiple opportunities to screen, counsel, and refer those at risk for negative outcomes. Primary care providers are also well-positioned to initiate behavioral counseling and referral as a result of long-term relationships they develop with adolescent patients. However, despite recommendations from professional organizations [5,6], screening and follow-up counseling in primary care remain inconsistent [3,7].

One strategy for increasing the frequency of screening and follow-up counseling for adolescent health risk behaviors in primary care is the use of electronic screening tools [8-13]. The benefits of electronic screening tools include their ability to streamline the screening process through branching logic and to generate reports to inform follow-up counseling by providers [14]. Electronic tools also provide an opportunity to deliver direct feedback to adolescents to motivate behavior change.

Whereas some electronic screening tools have incorporated feedback, educational components, or brief interventions [9,10], there remains limited data to understand how adolescent patients react to receiving such information. However, previous investigations of automated interventions for a range of age groups do provide an initial indication of specific feedback components that may effectively promote health behavior change. For adults, feedback strategies that raise awareness of health risks and promote self-efficacy to change have been shown to be effective in increasing physical activity and changing nutrition behavior [15]. Among college students, feedback that frames behavior in the context of their peers has been shown to reduce alcohol use and risky sexual behaviors [16-18]. Among adolescents, brief Web-based interventions that include elements intended to raise awareness of health risks, promote self-efficacy, and compare behavior with peer norms have produced small effects on drinking, though data suggests that reductions in alcohol use were primarily associated with raising awareness of health risks and promoting self-efficacy [19,20]. To our knowledge, few studies have examined the use of feedback for adolescent health behaviors outside of alcohol use or for multiple risk behaviors, and with some exceptions [21], few studies have sought direct adolescent reactions to receiving such information following screening.

In this study, we used qualitative methodology to better understand youth perceptions of and preferences for receiving feedback by evaluating their responses to an electronic screening tool for primary care (the “Check Yourself” tool) that incorporates immediate, personalized feedback for multiple health risk behaviors and reinforcement for health promoting behaviors. Adopting a similar approach to previous qualitative investigations of electronic health tools [22-26], we conducted individual semistructured interviews exploring youths’ overall impressions of the Check Yourself tool and their perceptions regarding the types of feedback messages that most motivated them to consider healthy behaviors, as well as what additional features would be helpful. In recognition of both the strengths and limitations of qualitative methodology, we aimed not to derive generalizable conclusions about the electronic health feedback delivered by the tool but to provide general suggestions for future feedback message development. Additionally, this qualitative data will supplement future quantitative outcomes from several randomized controlled trials of the tool currently being conducted by our research group.

## Methods

### Recruitment and Participants

Participants were recruited primarily from an adolescent-focused, academic clinic in Seattle, WA using a purposive sampling approach [27]. We selectively approached potential participants in the waiting area of the clinic to recruit a sample with approximately equal numbers of males and females and 13-15 and 16-18 year olds. Regarding race and ethnicity, we aimed to draw a sample representative of the Seattle area. Some participants were also recruited through flyers and word of mouth. Purposive sampling is preferred in qualitative research as it promotes the collection of a range of perspectives.

Adolescents were eligible to participate if they were in the age range of 13-18 (inclusive) years and could read and speak English. For participants aged 13-17 years, both youth assent and parental consent was obtained. For participants aged 18 years, youth consent was obtained, and parental consent was not required. Recruitment continued until theoretical saturation was reached (see “Data analysis” section below).

### Procedure

Adolescents first completed the Check Yourself tool and subsequently participated in individual qualitative interviews. The individual interview format was used to protect confidentiality and to allow interviewers time to explore the complexities of each participant’s perspectives. There were three interviewers in total (GZ, KK, and one other interviewer), all of whom completed training in qualitative methods before conducting interviews. Interviews occurred primarily in a private room in the same building as the adolescent clinic used for recruitment, at a time convenient for participants. When logistical constraints did not allow for the interview to occur at the clinic, interviewers met participants in a convenient and private location (eg, a private meeting room at a library). A majority of the interviews lasted 60 min (range: 45-90 min). Interviews occurred from February to July, 2015. Interviewers were gender-matched with participants as much as possible to promote comfort for participants in discussing sensitive health topics. Before the beginning of the interview, interviewers explained to participants that they did not personally develop the tool and that negative feedback was as valuable as positive feedback. Participants were oriented in this way to ensure that they did not suppress negative feedback to please the interviewer. Additionally, the interview guide contained several questions designed to elicit adolescents’ suggestions regarding how the tool could be improved. Adolescents received US $30 for participating. All study procedures were approved by the institutional review board of Seattle Children’s Research Institute.

**Figure 1:**
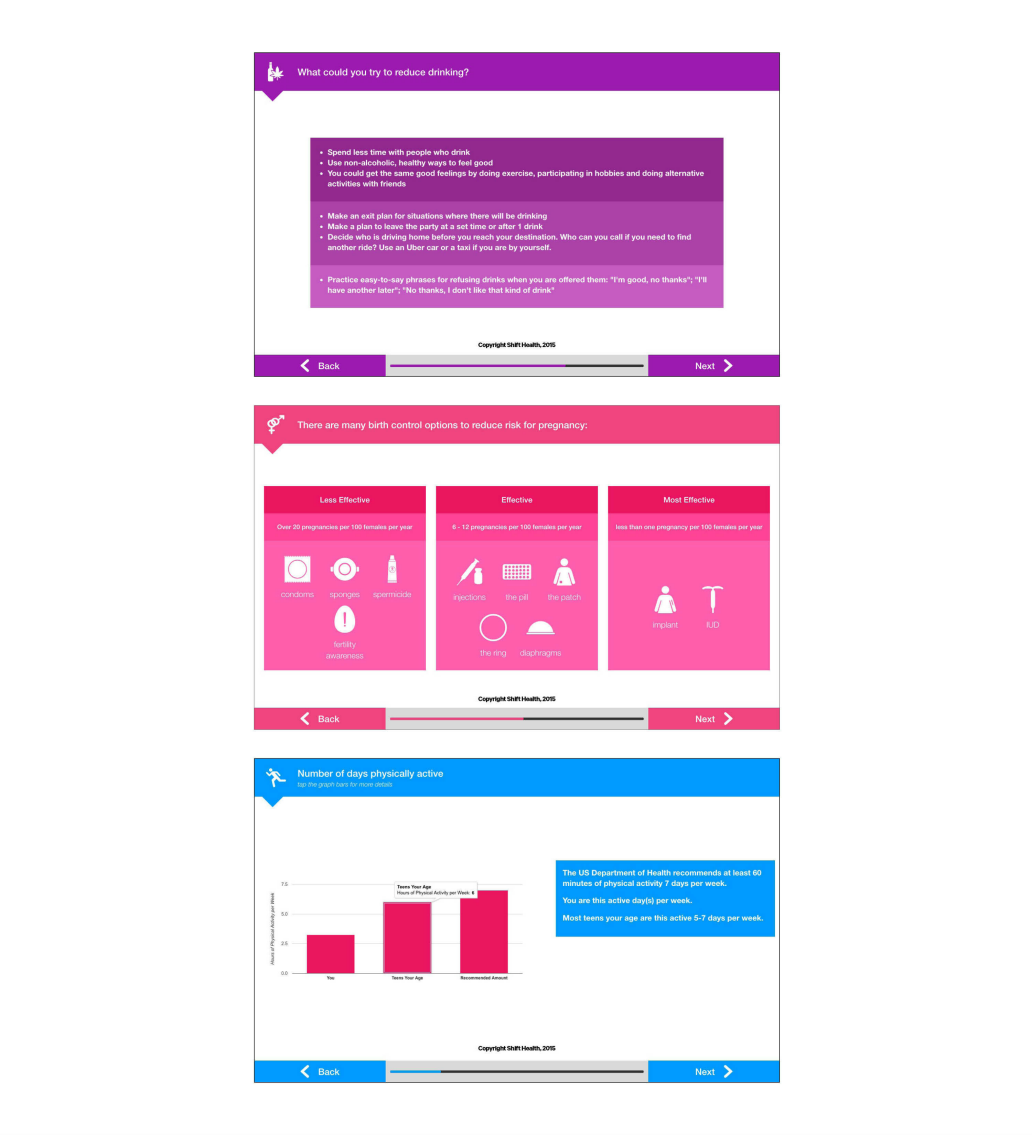
Example screenshots from the Check Yourself tool.

### The Check Yourself Tool

The Check Yourself tool is a tablet-based screening instrument developed by researchers at the University of Washington (LR and CM) in conjunction with the digital health company Shift Health [28], using TickiT, a platform for interactive patient-reported outcome measurement [29]. Designed with the goal of enhancing patient-provider communication, the tool features accessible language, colorful graphics, and integrated feedback based on screening responses. Assessment questions were developed based on screening recommendations from Bright Futures and the Guidelines for Adolescent Preventive Services [30,31], querying youth about eating and nutrition, exercise, screen time, sleep, safety behaviors (eg, wearing seatbelts and texting [short message service, SMS] while driving), drug and alcohol use, sexual behavior, and emotional health. The personalized feedback was informed by strategies shown to be effective by research on motivational interviewing [32] and specifically included information about (1) how adolescents’ responses compared with normative peer data and national health guidelines, (2) benefits and consequences of engaging in behaviors, (3) educational information about health choices, and (4) tips and suggestions for increasing healthy behaviors. Adolescents received this feedback immediately after completing the assessment section (see Figure 1 for example screenshots). The Check Yourself tool requires approximately 15 min to complete.

### Interview Content

Semistructured interviews were conducted using an interview guide that covered three areas (Textbox 1). As previous work has supported the usability of the screening segment of the tool [29,33], the interview guide for this study aimed primarily to elicit adolescent’s perspectives regarding the content of the feedback messages.

**Items from qualitative interview guide by topic.**

Overall impressions of the Check Yourself tool

- What was your overall experience with the tool?
- When you used the tool, did you find any parts confusing? Which parts?

Perceptions of motivational feedback

- Which health messages made the biggest impression on you?
- How did using the tool make you feel about your own health?
- Was there anything you thought you would want to change after using the tool?
- Which messages made you feel motivated to change?
- What additional information could have helped you feel more motivated?

Desired expansions of tool

- Are there other things you think we should add to the tool that would be helpful?
- Do you think it would be helpful if the tool sent you follow-up resources and websites? What would be most helpful to send?

### Data Analysis

Interviews were audio recorded and transcribed. Interview transcripts were uploaded to Dedoose, a Web-based qualitative analysis platform [34] and independently coded by two analysts. Coding discrepancies were resolved by consensus. A codebook was developed based on the interview guide and iteratively refined as transcripts were coded. When necessary, transcripts were recoded to reflect the current state of the codebook. Following the method of thematic analysis [35], the authors collaboratively reviewed all text excerpts within each code to identify both themes expressed across participants and key quotes which best illustrated each theme. An inductive approach was taken in the thematic analysis as the authors did not investigate prespecified themes but rather let participants’ responses guide the identification of themes. When selecting illustrative quotes, we prioritized quotes that (1) gave a full and articulate expression of a theme, and (2) which remained easily interpretable without the context of the full interview transcript. When data saturation occurred, defined in this study as no new themes being generated across three consecutive interviews, participant recruitment was discontinued.

Following Hill et al [36], themes are presented below with regular terms to indicate the level of agreement between participants. “All participants” or “in general” indicates agreement across all or almost all participants. “Many participants” indicates agreement across approximately half of participants. “Some participants” indicates agreement across a few participants.

## Results

### Participant Recruitment and Characteristics

In total, 26 participants were recruited from the waiting area of the clinic, 5 through word of mouth, and 1 via flyer. In total, 32 interviews were conducted, though 1 interview was not analyzed as the participant provided predominantly single-word responses that were not felt to enhance understanding of the youth’s perspective. The final sample therefore consisted of 31 adolescents. Participants’ demographic data are displayed in Table 1.

**Table 1:** Participant demographic data (n=31).

Characteristic	Value	Participants
Age (years), mean (SD^a^)		15.2 (1.4)
**Age (years), n (%)**		
	13	5 (16)
	14	5 (16)
	15	6 (19)
	16	10 (32)
	17	4 (13)
	18	1 (3)
**Gender, n (%)**		
	Male	13 (42)
	Female	18 (58)
**Ethnicity, n (%)**		
	Hispanic	6 (19)
	Non-Hispanic	25 (81)
**Race, n (%)**		
	African American	2 (7)
	Asian	3 (10)
	White	20 (65)
	Multiracial	2 (7)
	Race not specified	4 (13)

SD: standard deviation.

### Qualitative Results

Themes are presented below and grouped by the three topic areas of the interview guide: (1) overall impressions of the Check Yourself tool, (2) reactions to the personalized feedback, and (3) desired expansions of the tool. These topic areas are not themes themselves but rather broader categories of themes.

#### Overall Impression of the Check Yourself Tool

In general, participants reported that the Check Yourself tool was easy to use and that colorful images and interactive content increased their interest in the health information that was presented:

Teenagers, we like color...Bright colors make it fun, make it not like your filling out paperwork at a hospital or a clinic.

Female, 18

The true and false questions at the end, or at least it was when I did it. Which was a good memory thing because when I went through it...there I had to answer those things so I actually remembered them.

Male, 15

All participants indicated that they would prefer the Check Yourself tool to pencil-and-paper screening. Some adolescents particularly appreciated that questions were presented one at a time such that responses to previous questions were not visible. They felt this feature would help conceal their responses from family members in a waiting area:

I didn’t want anyone else to see [my responses] because my sister was sitting here, and my mom was sitting here...but this way like I said before was kind of like, you can answer the question and quickly move on and no one will see your answer if you do it fast enough.

Female, 16

Some participants also noted that not displaying responses to previous questions would make it less upsetting to endorse risk behaviors since they didn’t have to continue to see their responses as they waited to see their provider.

Adolescents described distinct ways that they felt the Check Yourself tool could enhance their interactions with doctors. Some thought the tool could function as a “warm up before the main event” of an appointment by priming them to identify their questions and concerns to discuss during the visit. Many participants found it easier and less awkward to disclose health risk behaviors on the tool than face-to-face, and perceived the tool as helpful in reducing providers’ need to ask patients about sensitive topics during the appointment:

I like the idea of having [the tool] because a lot of people, like I know a lot of times I would go to the doctor ready to say something and then get scared and not say it. This way, it’s a little bit impersonal, but at least I’m getting it down and so the doctor, I wouldn’t have to make eye contact with him, but he will know because I put it in there.

Female, 16

#### Responses to the Personalized Feedback

Adolescents commented on several aspects of the personalized feedback provided by the Check Yourself tool. In general, participants stated that the tool provided new information including education, tips for health improvement, and information on how their behavior compared with peer norms and national guidelines for adolescent health. Nearly all youth appreciated the presentation of information in a nonjudgmental manner:

It wasn’t super forceful. It was kind of like here’s an amount that you eat, and here’s the amount that people your age normally eat, and here’s the recommended amount...I like how it wasn’t super forceful like black screen, red words, eat more fruits and vegetables.

Female, 18

The most commented on feedback components were the graphs which compared the participants’ own responses with peer norms and national health guidelines. Many reported that they found these comparisons to be motivating:

When I saw the graph of the physical activity, I was way below, and I was just thinking, “Wow, I really should do something about that.”

Male, 14

However, some youth felt that the comparisons were not helpful. The most common concern regarding comparisons to peer norms was that the statistics were not accurate or relevant as they were not consistent with what participants encountered in their close friend groups:

When I was looking at some of the numbers I was like, ‘This doesn’t seem right’ because—or at least my high school it might seem different from what I see around me. I felt like some of those [numbers] might have been a little low, the numbers of the average teen.

Female, 17

Even if no one else in the school [used marijuana] but my friends, it wouldn’t exactly matter to me because people I know do—that’s what’s relevant.

Female, 14

Regarding comparisons with national health guidelines, some adolescents disagreed that recommendations for certain behaviors (eg, screen time) were any healthier than what they were already doing. Participants typically voiced this objection if they had not yet experienced any consequences from their behaviors:

Personally, I’m happy with what I do right now. I have a little bit too much screen time, but I sleep eight hours every night, I do cross country, track, and winter running club...The consequences that [the tool] mentioned were loss of sleep, less time to do athletic stuff, and things like that which were all things that it previously said I was great with.

Male, 15

Sometimes, even if participants recognized that following the health guidelines would be beneficial, they perceived the recommendations as “unreasonable” in the context of modern adolescent lifestyles. This objection was voiced primarily in relation to screen time and exercise recommendations (<2 hours/day and 1 hour, 7 days/week, respectively). Lastly, some participants found the comparisons too stark and suggested that it would be helpful to add validating phrases to soften the message:

The slides that were specifically around nutrition and exercise, especially when you’re not meeting it, it’s very jarring to be like, “You’re not meeting it,” and there’s no kind of soft landing that you get when you talk to a person and they’re like, “Well, you’re doing a pretty good job,” and some of the little changes you need to make to get there.

Male, 17

In addition to normative comparisons, many participants reported that they were motivated by learning about the benefits of healthy behaviors:

When I did see that cause and effect thing it kind of made me think, “Well, that effect would be nice.”

Female, 17

Some others felt that learning about the consequences of risk behaviors was more motivating and specifically requested more alarming statistics. It is worth noting, however, that those endorsing this view tended to not be engaging in risk behaviors themselves and were more often reflecting on what they felt would be motivating for their peers who were.

Many adolescents also perceived value in aspects of the feedback which aimed to promote self-efficacy for and commitment to behavior change, including practical tips to change behaviors. Many were interested in getting tips regardless of whether they were engaging in risk behaviors, as the tips might be useful in the future:

Back to the sexual activity and stuff it gave a feedback, like stages of what is better to use like for birth control-wise...Eventually I’m going to be sexually active and I want to plan what happens and what I should use and I want to be safe.

Female, 15

Participants reported that the tips regarding how to increase and sustain healthy behaviors, as well as how to stay safe if engaging in risk behaviors (eg, ways to drink alcohol responsibly) were all useful. Finally, many adolescents appreciated validation for healthy behaviors:

I think just affirming the fact that—for instance, I put down that I always wear seat belts...It’s nice to be like “Yeah, that’s the right thing to do.”

Male, 17

#### Desired Expansions of the Tool

Participants expressed interest in four potential expansions for the Check Yourself tool: receiving follow-up information, goal-setting, tracking of behaviors over time, and communication with providers in between visits. Regarding follow-up content, many desired more information about specific health behaviors for personal research and learning and thought that it would be helpful if the tool would provide a targeted set of links and resources for areas of interest. Many adolescents were also interested in additional resources for changing behaviors, especially practical “tips” (eg, for how to start going to bed earlier).

Many adolescents commented that they would like to have the opportunity to set goals based on the feedback they received from the Check Yourself tool. In talking about goal setting, participants expressed the importance of being able to determine their own goals and desired a process that emphasizes small steps and integrates with follow-up information:

Maybe there could be an option for their goal in each section like at the end of [the tool]...I think if it’s a goal that could help with sent out information—so if they say their goal is to get two more hours of sleep every night, we could get information about the best ways to do that.

Female, 14

I would say have little steps in there to getting better. Take steps—baby steps—to getting better.

Male, 15

Many participants also wanted to expand the tool to include tracking health behaviors over time and in relation to goals. Some thought that tracking systems would ideally be more integrated and able to recognize and alert them to patterns across health behaviors (eg, relationships between physical activity and eating). As a part of ongoing tracking, some participants indicated an interest in receiving electronic reminders and ongoing motivational messaging about goals that they had set:

I guess [it would be useful to send] just how often I should do it just as a reminder and also encouragement as to why I wanted to do it in the first place.

Female, 14

Notably, some participants indicated a preference for electronic reminders over reminders from parents or other adults, particularly in the context of goals that they had set for themselves:

Those things where your parents would probably remind you, but your parents are like, “Hmm,” but then [with electronic reminders] it’s like oh maybe I should do it because it’s good for me and it’s me doing it, not my parent to be like “Go do it.”

Male, 17

Some adolescents described a desire for their providers to be involved in the behavior tracking process, specifically wanting doctors to view their progress in between visits and to provide encouraging comments.

## Discussion

### Principal Findings

In this qualitative study, we examined adolescents’ perspectives on an electronic health screening tool for primary care settings that provides personalized feedback. Overall, the tool was well-received by participants, who strongly preferred electronic screening over paper-and-pencil forms. Youth appreciated the colorful and interactive content, valued aspects of the tool that enhanced privacy, and indicated that they would disclose more health risk behaviors to the tool than to paper-and-pencil forms, consistent with prior research on electronic health screening [29,37-41]. Importantly, and also consistent with prior research on electronic screening, participants perceived the tool as a way to enhance—but not replace—interactions with providers by helping them to identify questions and concerns before an appointment [41,42]. To date, studies regarding screening instruments have focused primarily on the frequency of provider counseling and referral [8-12]. However, our data suggest that electronic screening with feedback could be used to impact adolescent behavior during the appointment. For example, screening and feedback may increase adolescent interest in discussing health concerns with providers. Patient communication behaviors such as these have been linked with positive health outcomes [43,44]. Future research should investigate the possibility that electronic screening with feedback influences adolescent behavior during the appointment.

Adolescents thought that the feedback delivered by the tool was generally useful and motivating. They appreciated that the feedback was presented with nonjudgmental language and responded positively to a variety of specific feedback components including information regarding the benefits of healthy behaviors, risks of negative behaviors, tips for behavior change, and the reinforcement of good choices.

Whereas many found the comparisons with peer norms and national health guidelines interesting and helpful, a small but noteworthy minority of participants dismissed the peer data as not real or relevant and guidelines as not valid or reasonable. The perspectives expressed by this minority suggest that adolescents are discerning consumers of peer normative data and that peer comparisons should be presented in a way that enhances the perceived relevance and credibility of the information. Additionally, participants in this study could have responded more truthfully about their health risk behaviors than adolescents in national survey research, spuriously making participants’ behaviors appear worse than the national averages. Whereas more data is needed to evaluate the possibility that adolescents respond more honestly to electronic health screening tools than to anonymous national survey research, future interventions including normative feedback should consider the limitations of comparing self-reported health data gathered through different modalities.

In any case, when presenting normative feedback, it may be important to select comparison groups as similar as possible to individual adolescents (eg, with respect to age, gender, and school) and to clearly cite data sources to increase perceptions of relevance and credibility. Notably, the two studies which have investigated Web-based interventions, including normative feedback for adolescent alcohol use, either did not present school specific norms or did not present age and gender specific norms, and did not find a connection between normative feedback and reductions in alcohol use [19,20]. In contrast, electronic normative feedback interventions for college students that have incorporated norms specific to age, gender, and campus have been associated with significant reductions in alcohol use and risky sexual behavior [16-18]. In sum, normative misperceptions are prevalent among adolescents and contribute to a variety of health risk behaviors in addition to alcohol and drug use [45]. In order to correct these misperceptions, targeted data presentation techniques are needed to ensure that adolescents trust and believe normative feedback offered to them.

Regarding comparisons with national health guidelines, some participants specifically found the recommendations for screen time (<2 hours/day) and exercise (1 hour, 7 days/week) unreasonable in the context of their daily lives. The media guidelines used in the Check Yourself tool were based on the 2013 guidelines from the American Academy of Pediatrics [46] that were recently updated in recognition of the increasing integration of technology into modern adolescent lifestyles [47,48]. The 2016 guidelines emphasize individualized family planning of appropriate media use and lean away from strict quantitative cutoffs [49]. Future studies should examine if the new guidelines are more acceptable to youth. Whereas the exercise recommendations were also viewed as difficult to achieve by adolescents in our study, these guidelines are based on extensive empirical evidence [50]. However, our data suggest that presenting what adolescents may perceive as stringent exercise guidelines may not promote motivation to change unless paired with strategies to promote self-efficacy such as practical tips and suggestions to set small achievable goals.

When prompted with possible adaptations to the tool, adolescents expressed interest in receiving follow-up information about health risks, opportunities to set goals and track health behaviors, receiving reminders of planned changes, and communicating with providers electronically between appointments. Participants’ enthusiasm for these additional features suggests that adolescents may envision an ongoing role for technology in health behavior change, and specifically technological supports that enhance their ability to self-regulate behaviors and include the option of seeking professional input if desired. These findings corroborate results from other recent qualitative studies investigating adolescents’ preferences for text messaging-based preventive interventions, which have documented high youth interest in receiving brief, personalized advice and reminders of reasons to change on an ongoing basis [26,51]. Taken together, adolescents’ interest in highly personalized content lends additional support to increasing evidence that Web-based interventions incorporating tailored information yield stronger effects than generic interventions [52]. Whereas such technological supports should address the needs and preferences of adolescents, care must also be taken to ensure that busy primary care providers have tools and methods to rapidly interpret and utilize adolescent-generated data. Future research could address this issue.

### Limitations

The qualitative design of this study entails both strengths and limitations. Though we were able to examine nuanced perspectives not easily accessible to quantitative research, it is possible that adolescents’ opinions regarding effective types of feedback expressed during a qualitative interview may not reflect what would actually have an impact if tested using quantitative methodology. For this reason, results from this study should function to inform hypothesis generation rather than generalizable knowledge. An additional limitation stems from our recruitment from primarily one clinic in Seattle, WA, which resulted in a sample largely representative of this geographic region but whose perspectives may not describe those of adolescents in other areas and health care settings.

### Conclusions

With the opportunity to create screening tools that go beyond collecting information from adolescent patients by providing education and feedback, it is useful to seek youth input in order to design content that is acceptable and effective for this population. Adolescents in this qualitative study were specific about their preferences for electronic, personalized feedback on their health behaviors. They expressed engagement with and enthusiasm for the process of receiving feedback in general and were appreciative of new health information and tips, while at the same time they offered some critiques and suggestions regarding specific feedback messages. Participants desired feedback that validates healthy behavior choices and supports them as independent decision makers by neutrally presenting relevant information. Additionally, participants valued feedback that enhances their ability for self-management by facilitating goal setting and offering ongoing technological supports. Future quantitative outcome research should test screening tools that incorporate these suggestions.
